# Influence of KIR and NK Cell Reconstitution in the Outcomes of Hematopoietic Stem Cell Transplantation

**DOI:** 10.3389/fimmu.2020.02022

**Published:** 2020-09-02

**Authors:** Fei Gao, Yishan Ye, Yang Gao, He Huang, Yanmin Zhao

**Affiliations:** ^1^Bone Marrow Transplantation Center, The First Affiliated Hospital, School of Medicine, Zhejiang University, Hangzhou, China; ^2^Institute of Hematology, Zhejiang University, Hangzhou, China; ^3^Zhejiang Engineering Laboratory for Stem Cell and Immunotherapy, Hangzhou, China

**Keywords:** KIR, NK cell reconstitution, hematopoietic stem cell transplantation, GVHD, infection, relapse

## Abstract

Natural killer (NK) cells play a significant role in immune tolerance and immune surveillance. Killer immunoglobin-like receptors (KIRs), which recognize human leukocyte antigen (HLA) class I molecules, are particularly important for NK cell functions. Previous studies have suggested that, in the setting of hematopoietic stem cell transplantation (HSCT), alloreactive NK cells from the donor could efficiently eliminate recipient tumor cells and the residual immune cells. Subsequently, several clinical models were established to determine the optimal donors who would exhibit a graft-vs. -leukemia (GVL) effect without developing graft-vs. -host disease (GVHD). In addition, hypotheses about specific beneficial receptor-ligand pairs and KIR genes have been raised and the favorable effects of alloreactive NK cells are being investigated. Moreover, with a deeper understanding of the process of NK cell reconstitution post-HSCT, new factors involved in this process and the defects of previous models have been observed. In this review, we summarize the most relevant literatures about the impact of NK cell alloreactivity on transplant outcomes and the factors affecting NK cell reconstitution.

## Introduction

Allogeneic hematopoietic stem cell transplantation (allo-HSCT) is an effective therapy for patients with hematological malignancies. However, relapse, graft-vs. -host disease (GVHD), and infections remain the main causes of treatment failure (1–4). Potential strategies to prevent GVHD and even infections while sparing the graft-vs. -leukemia (GVL) effect have attracted extensive attention. Natural killer (NK) cells, which are a major type of innate lymphocytes, are being researched in this context.

NK cells constitute 5–15% of human peripheral blood lymphocytes (5, 6) and possess the abilities of cytotoxic lysis and rapid cytokine secretion without prior antigen presentation (7, 8). These functions are regulated by various types of receptors expressed on NK cells manifesting multiple functions either activating or inhibitory (9–11) (Table 1). Among the NK cell receptors, the killer immunoglobin-like receptor (KIR) is one of the major factors that mediate self-tolerance and anti-tumor/infection responses.

**Table 1:** NK cell receptors and their ligands.

**Inhibitory receptors and their ligands**		**Activating receptors and their ligands**		**Coreceptors and their ligands**	
KIR2DL1	HLA-C2	KIR2DS1	HLA-C2	2B4	CD48
KIR2DL2	HLA-C1	KIR2DS2	HLA-C1	NTB-A	NTB-A
KIR2DL3	HLA-C1	KIR2DS3	Unknown	CS1	CS1
KIR2DL4	HLA-G	KIR2DS4	HLA-A11	NKp80	AICL
KIR2DL5	Unknown	KIR2DS5	Unknown	TLR	TLRL
KIR3DL1	HLA-Bw4	KIR3DS1	HLA-F	DNM-1	PVR, Netcin-2
KIR3DL2	HLA-A3/A11	NKG2C	HLA-E	CD96	PVR
KIR3DL3	Unknown	NKG2D	MICA, MICB, ULBP1-4		
NKG2A	HLA-E	NKp30	B7-H6, BAT3, CMV pp65		
LIR-1	HLA class I	NKp44	Viral hemagglutinins		
		NKp46	Viral hemagglutinins		
		CD16	IgG-1, 3, 4		

**Figure 1:**
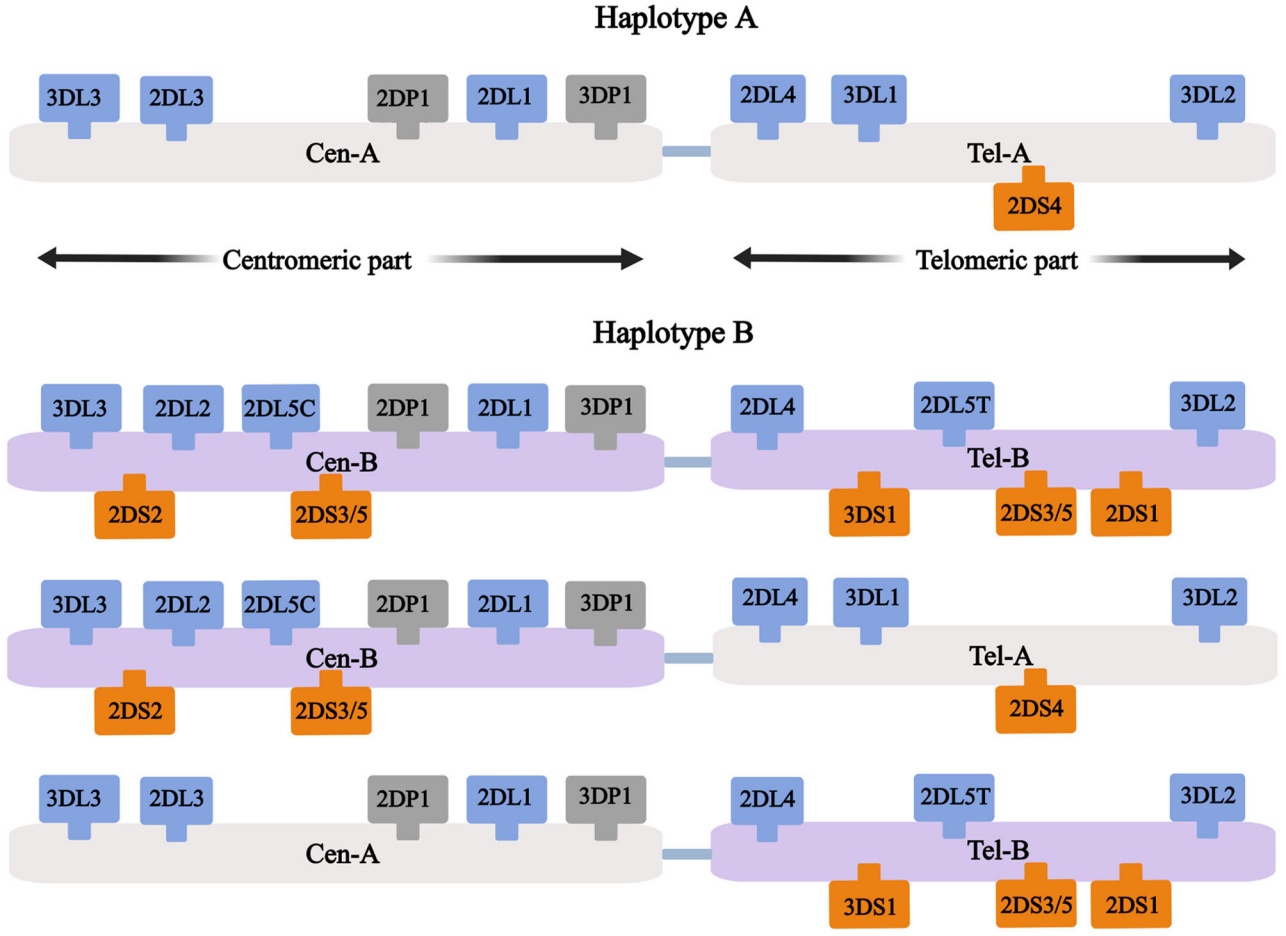
Simplified genomic maps of KIR. Inhibitory KIR genes are color-coded in blue, activating KIR genes in orange, and pseudogenes in gray. KIR haplotype A has only one activating KIR gene: KIR2DS4, KIR B haplotype has fixable content of activating KIR genes. KIR haplotype could be further determined as Cen haplotype and Tel haplotype.

It is well established that KIR genes are located on chromosome 19q13.4 (12). Based on their various structures (the number of extracellular immunoglobulin domains (D) and the long (L) or short (S) tails), 16 KIR genes (including two pseudogenes (P), KIR2DP1 and KIR3DP1) have been classified into four groups (KIR2DL1-5, KIR3DL1-3, KIR2DS1-5, and KIR3DS1). Six genes with short tails are activating KIR genes that encode activating receptors, while the eight genes with long tails are inhibitory KIR genes encoding inhibitory receptors. KIRs could be divided into haplotype A and B according to the activating genes on them. Haplotype A has only one activating gene, KIR2DS4, whereas haplotype B possesses up to five activating KIR genes, including KIR2DS1, 2, 3, 5, and 3DS1 (Figure 1). Thus, the A/A genotype is defined as homozygous for A haplotypes, and the B/x genotype consists of at least one B haplotype. Finally, according to the specific KIR gene locus on the chromosome, a centromeric (Cen) and telomeric (Tel) KIR haplotype and genotype are further determined (13–15). Five inhibitory and three activating KIRs recognize specific class I HLA (A, B, or C) ligands, with the inhibitory KIR2DL1 recognizes group 2 HLA-C alleles, KIR2DL2 and KIR2DL3 recognize group 1 HLA-C alleles, KIR3DL1 recognizes HLA-Bw4 alleles, and KIR3DL2 recognizes HLA-A3/-A11 alleles. Moreover, activating KIR2DS1, KIR2DS2, and KIR2DS4 recognize HLA-C2, C1, A11, respectively (15). The ligands of the remaining KIRs remain unknown.

**Figure 2:**
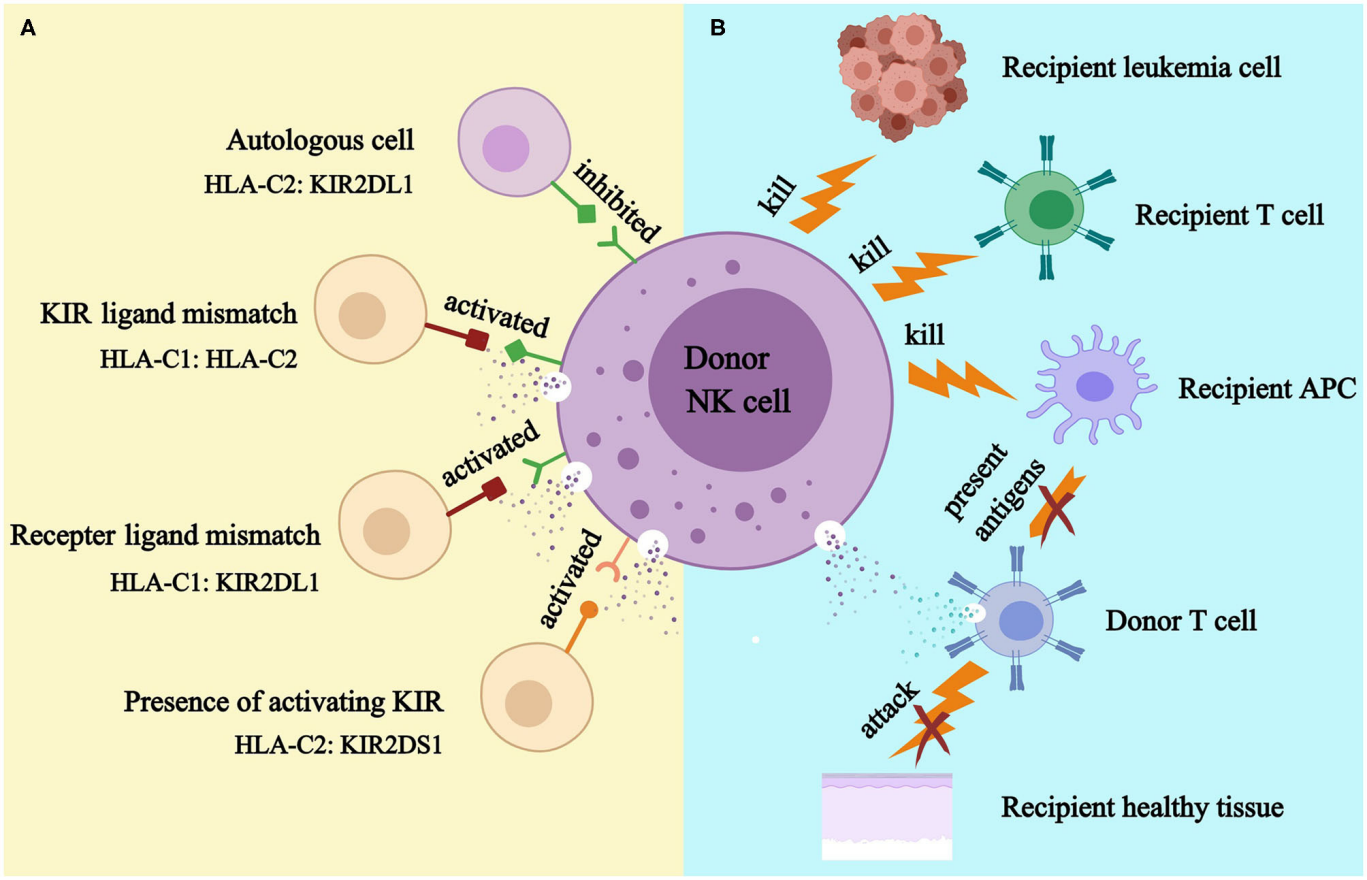
KIR models **(A)** and NK cell-mediated killing **(B)**. APC, antigen presenting cell. **(A)**. Donor NK cell is tolerant to self because donor inhibitory KIR is inhibited by its cognate HLA ligand; donor NK cell might kill recipient cell because HLA ligand for donor inhibitory KIR presents in donor but absents in recipient (KIR ligand model); donor NK cell could kill recipient cell because recipient HLA ligand does not inhibit donor inhibitory KIR (receptor ligand model); donor NK cell could kill recipient cell because donor activating KIR is activated by recipient (KIR B haplotypes and KIR B genes). **(B)**. Alloreactive donor NK cell could kill recipient leukemia cell to prevent relapse; it could kill recipient T cell to prevent graft rejection; and it could kill recipient APC to prevent GVHD.

As KIR genes and human leukocyte antigen (HLA) genes are located on different chromosomes, autologous KIR receptor-ligand mismatch may exist (16). Normally, NK cells acquire self-tolerance and functional competence through the education process, in which inhibitory KIRs could be inhibited by self-HLA ligands and activated in a non-self HLA environment. Besides, the decreased responsiveness of activating KIRs in the presence of their cognate ligands also prevents autoimmunity (17–23) (Figure 2A). Importantly, infected and/or tumor cells may express inhibitory KIR ligands insufficiently or express activating ligands that may activate NK cells (24–31).

As the first reconstituted lymphocyte subset after transplantation (32, 33), NK cells play a critical role in controlling early relapse and infections. They also possess the ability to eliminate recipient T cells and antigen-presenting cells (APCs), to prevent graft failure and GVHD (34–38) (Figure 2B). Three models were established historically in an attempt to optimize donor selection for HSCT based on KIR (Figure 2A). The Perugia group in Italy firstly proposed the donor-recipient KIR ligand-ligand model (also known as KIR ligand model) solely based on the HLA phenotype of the donor and recipient. The KIR ligand incompatibility in the GVH direction was defined as the absence in recipients of donor class I allele group(s) recognized by KIRs. Those authors observed that the HLA haplotype-mismatched transplants reduced the rejection and relapse rate and prevented GVHD in patients with acute myeloid leukemia (AML) (36). Subsequently, the second model (named receptor-ligand model or missing ligand model) was raised by Leung et al. based on the compatibilities between the recipient HLA and donor inhibitory KIR. This model focused on donor KIR instead of donor HLA and could, therefore, be used in both HLA-matched and HLA-mismatched transplants. The results of that study suggested that the receptor-ligand model better predicted the risk of primary disease relapse, especially for lymphoid malignancies, compared with the ligand-ligand model (39). Subsequently, with a deeper understanding of KIR haplotypes, the third model analyzed and compared the KIR genotypes of different donors. Cooley et al. showed that unrelated donors with KIR-B haplotypes conferred a significant relapse-free survival (RFS) benefit to patients with AML undergoing T cell-replete HSCT (40). Based on the three models described above, numerous studies have been carried out to explore the impact of NK cell alloreactivity. Clinical results obtained from KIR ligand model, receptor ligand model and KIR haplotype and gene model were summarized in Tables 2–4, respectively. Nevertheless, the results were controversial, and several key questions remained regarding NK cell biology post-HSCT. What are the exact effects of NK cell alloreactivity on patients after HSCT? How do NK cells reconstitute post-HSCT and which factors may interfere with the reconstitution process? This review summarizes the latest literature on this important topic and offer some instructive hypothesis.

**Table 2:** Impact of KIR on clinical outcomes in KIR ligand model.

**References**	**N**	**Disease**	**Donor**	**Graft manipulation**	**Clinical outcomes**
Ruggeri et al. (36)	92	AML, ALL	HRD	TCD^*^	KIR ligand mismatch: higher EFS and OS, lower relapse (AML) KIR ligand mismatch: lower aGVHD^2−4^
Davies et al. (41)	175	Mixed	URD	TCD^*^, TCR	KIR ligand mismatch: lower OS (myeloid cohort)
Giebel et al. (42)	130	Mixed	URD	TCD#	KIR ligand mismatch: higher OS and DFS, lower TRM
Schaffer et al. (43)	190	Mixed	URD	TCD^*^, TCD#	KIR ligand mismatch: higher IRM and TRM, and lower OS
Elmaagacli et al. (44)	236	CML	MSD, URD	TCR	KIR ligand mismatch: lower molecular relapse
Yabe et al. (45)	1489	Mixed	URD	TCD#, TCR	KIR ligand mismatch: higher aGVHD^2/3−4^ and lower OS (HLA-C mismatched transplants)
Verneris et al. (46)	716	Pediatric AL	URD	TCD#, TCR	KIR ligand mismatch: no significant impact on OS, DFS, relapse, TRM, or aGVHD.
Ruggeri et al. (47)	112	AML	HRD	TCD^*^	KIR ligand mismatch: lower relapse (CR group), higher EFS, and lower risk of relapse or death
Huang et al. (48)	116	Mixed	HRD	TCD#	KIR ligand mismatch: higher aGVHD^2−4^ and relapse, lower OS
Zhao et al. (49)	64	Mixed	HRD	TCD#	KIR ligand mismatch: higher aGVHD;
Michaelis et al. (50)	57	Mixed	HRD	TCD^*^	KIR ligand mismatch: lower EFS (AML)
Mancusi et al. (51)	161	AML, ALL	HRD	TCD^*^ TCD^*^+Treg/Tcon	NK-alloreactive donors: lower relapse and higher EFS (AML)
Yahng et al. (52)	100	AML	HRD	TCD#	KIR ligand mismatch (HVG): higher relapse and CMV reactivation, lower DFS
Zhao et al. (53)	180	Mixed	HRD	TCD#	KIR ligand match: lower CMV reactivation rate and higher IFN-γ expression
Wanquet et al. (54)	144	Mixed	HRD	TCD#	KIR ligand mismatch: lower relapse and higher PFS (no CR group)
Shimoni et al. (55)	444	AML, ALL	HRD	TCD#	KIR ligand mismatch: a trend of higher relapse (AML), lower OS

*MSD, matched sibling donor; URD, unrelated donor; HRD, haploidentical related donor; AML, acute myeloid leukemia; ALL, acute lymphoid leukemia; CML, chronic myeloid leukemia; TCD, T cell depleted; TCR: T cell replete; Treg, regulatory T cells; Tcon, conventional T cells; aGVHD: acute graft vs. host disease; cGVHD: chronic graft vs. host disease; OS, overall survival; RFS, relapse free survival; DFS, disease free survival; EFS, event free survival; IRM: infection related mortality; TRM: transplant related mortality; CMV, cytomegalovirus*.

TCD^*^: ex-vivo TCD.

*TCD#: in-vivo TCD*.

**Table 3:** Impact of KIR on clinical outcomes in receptor ligand model.

**References**	**N**	**Disease**	**Donor**	**Graft manipulation**	**Clinical outcomes**
Leung et al. (39)	36	Mixed	HRD	TCD^*^	Receptor ligand mismatch: lower relapse
Cook et al. (56)	220	Mixed	MSD	/	HLA-C2C2 patients vs. HLA-C1/x patients: lower OS (myeloid cohort)
Verheyden et al. (57)	65	Mixed	MSD	TCD^*^, TCR	HLA-C1C2 patients vs. HLA-C1C1 or C2C2 patients: lower aGVHD
Hsu et al. (58)	1770	Mixed	URD	TCR	Missing ligand for donor iKIR: lower relapse (HLA mismatched transplants)
Clausen et al. (59)	43	Mixed	MSD	TCR	Ligand missing to KIR3DL2 plus one other iKIR vs. others: lower relapse and higher OS
Ludajic et al. (60)	124	Mixed	URD	TCD#, TCR	Missing ligand for donor KIR2DL1: higher aGVHD^2−4^;
Linn et al. (61)	151	Mixed	MSD	TCR	Missing ligand for donor iKIR: no impact on OS and RFS
Wu et al. (62)	48	Mixed	URD	TCD#	HLA group C1 vs. C2: higher CMV reactivation rate
Gagne et al. (63)	264	Mixed	URD	TCR	Missing HLA-C1 ligand: lower OS (myeloid cohort)
Clausen et al. (64)	100	Mixed	MSD	TCR	HLA-C1C2 patients vs. HLA-C1C1 or C2C2 patients: lower relapse and aGVHD^2−4^, higher RFS
Björklund et al. (65)	105	AML, MDS	MSD	TCD#, TCR	Receptor ligand mismatch: no significant impact on OS, relapse and GVHD
Wu et al. (66)	116	Mixed	URD	TCD#, TCR	Missing ligand for donor iKIR: lower relapse, higher OS and DFS (myeloid cohort);
Zhou et al. (67)	219	Mixed	MSD	/	HLA-C1C1 patients vs. HLA-C2/x patients: lower aGVHD^2−4^
Sobecks et al. (68)	909	AML, MDS	URD	TCD#, TCR	Missing ligand for donor iKIR: higher aGVHD^3−4^ and TRM (AML); Missing HLA-C2 for donor KIR2DL1: higher aGVHD^2/3−4^ (AML)
Park et al. (69)	59	Mixed	MSD, URD	TCD#, TCR	Receptor ligand mismatch: higher OS, DFS and lower relapse
Cardozo et al. (70)	50	Mixed	MSD	TCR	Patients with all ligands present vs. missing ligand for donor iKIR: higher aGVHD; Missing ligand for donor iKIR: higher OS (myeloid cohort)
Faridi et al. (71)	281	Mixed	MSD, URD	TCD#	Missing ligand for donor iKIR: lower relapse and better RFS (URD)
Neuchel et al. (72)	1446	Mixed	URD	TCR	HLA-C2C2 vs. HLA-C1/x patients: lower OS, DFS, higher relapse (myeloid cohort)
Arima et al. (73)	10638	Mixed	MSD, URD	TCD^*^, TCD# TCR	HLA-C1C1 patients vs. HLA-C1C2 patients: lower relapse and higher RFS (AML and CML); HLA-C1C1 patients vs. HLA-C1C2 patients: higher relapse (ALL)
Gaafar et al. (74)	87	Mixed	MSD	TCR	KIR2DL1: HLA-C2 match: higher aGVHD^2−4^ (AML)
Arima et al. (75)	2884	ALL	MSD, URD	TCD, TCR	HLA-C1C1 patients vs. HLA-C1C2 patients: higher relapse
Chen et al. (76)	84	Mixed	HRD	TCD#	Missing HLA-C2 ligand for donor KIR2DL1: higher OS and lower RRM (myeloid cohort); Missing HLA-C for donor iKIR: lower aGVHD^2−4^ (lymphoid cohort);
Zhao et al. (77)	97	CML	HRD	TCD#	Receptor ligand match: lower relapse
Zhao et al. (78)	188	Mixed	HRD	TCD#	Receptor ligand match: lower relapse and higher LFS
Solomon et al. (79)	208	Mixed	HRD	TCD#	Receptor ligand mismatch: higher OS and DFS, lower relapse
Willem et al. (80)	51	Mixed	HRD	TCD#	KIR2DL/HLA mismatch: higher GVHD and lower relapse

*AL, acute leukemia; MDS, myelodysplastic syndromes; iKIR, inhibitory KIR; LFS, leukemia-free survival*.

**Table 4:** Impact of KIR on clinical outcomes in KIR haplotype and gene model.

**References**	**N**	**Disease**	**Donor**	**Graft manipulation**	**Clinical outcomes**
Cooley et al. (40)	448	AML	URD	TCR	KIR B/x donor: higher RFS and cGVHD
Cook et al. (56)	220	Mixed	MSD	/	KIR2DS2: lower OS (HLA-C2C2 patients with myeloid diseases)
Verheyden et al. (57)	65	Mixed	MSD	TCD^*^, TCR	Donor co-presenting KIR2DS1 and 2DS2: lower relapse
Chen et al. (81)	131	Mixed	MSD	TCR	KIR2DS2: higher CMV reactivation (HLA-C2C2 patients); Additional activating KIR genes in donor: higher OS and lower CMV reactivation
Yabe et al. (45)	1489	Mixed	URD	TCD#, TCR	KIR2DS2: higher aGVHD^3−4^ (HLA-C mismatched transplants)
Schellekens et al. (82)	83	Mixed	MSD	TCR	KIR2DS1: higher OS (HLA-C1C1 patients); More activating KIRs in donor or patients: higher relapse; KIR2DS5 in patients or both in donor and patients: higher relapse
van der Meer et al. (83)	70	Mixed	MSD	TCD^*^	KIR2DS5: higher LFS and lower relapse (HLA-C1C1 or HLA-C2C2 patients); KIR2DS5: lower LFS and higher relapse (HLA-C1C2 patients)
Ludajic et al. (60)	124	Mixed	URD	TCD#, TCR	KIR2DS2: lower aGVHD^2−4^ (HLA-C1C2 patients)
Zaia et al. (84)	211	Mixed	MSD, URD	TCR	Donor co-presenting KIR 2DS2 and 2DS4: lower CMV reactivation; Donor aKIR gene content ≥5: lower CMV reactivation
Wu et al. (62)	48	Mixed	URD	TCD#	High aKIRs group: lower CMV reactivation rate
Gagne et al. (63)	264	Mixed	URD	TCR	KIR B/x donor: lower aGVHD^3−4^ (HLA identical pairs with myeloid disease)
Bao et al. (85)	75	Mixed	URD	TCD#	KIR B/x donor: higher OS
Venstrom et al. (86)	1087	Mixed	URD	TCD^*^, TCR	KIR3DS1: lower aGVHD^2−4^; KIR3DS1: lower aGVHD^2−4^, TRM and mortality (AML, CML and ALL)
Wu et al. (66)	116	Mixed	URD	TCD#, TCR	KIR2DS3: higher relapse, lower OS and DFS (myeloid cohort); More numbers of activating KIR genes in donor: higher relapse
Tomblyn et al. (87)	116	Mixed	URD	TCD^*^, TCR	KIR B/x donor: lower bacterial infections by day 180
Cooley et al. (88)	1409	AML, ALL	URD	TCR	KIR B/x donor: lower relapse and higher DFS (AML); Cen-BB vs. Cen-BA or AA: lower relapse and higher DFS (AML); Tel-B/x vs. Tel-AA: lower relapse (AML); B content ≥ 2: lower relapse (AML)
Venstrom et al. (89)	1277	AML	URD	TCD^*^, TCR	Donor KIR2DS1 with HLA-C1/x patients vs. with HLA-C2C2 patients: lower relapse; KIR3DS1: higher OS
Zhou et al. (67)	219	Mixed	MSD	/	Cen-B/x donor: higher OS, RFS and lower relapse
Impola et al. (90)	134	Mixed	MSD	/	KIR 2DL2 or KIR 2DS2: better RFS (AML)
Bao et al. (91)	210	Mixed	URD	TCD#	KIR B/x donor: higher OS, RFS and lower NRM (AML and MDS); Cen-B/x donor: higher OS, RFS (AML and MDS at standard risk)
Cardozo et al. (70)	50	Mixed	MSD	TCR	KIR2DS2: lower OS and EFS
Bachanova et al. (92)	614	NHL	URD	TCD#, TCR	KIR B/x donor: lower relapse and better PFS (HLA matched transplants)
Kamenaric et al. (93)	111	Mixed	MSD, URD	TCD#	KIR2DS4 (neg vs. pos): no impact on GVHD (MSD)
Hosokai et al. (94)	106	Mixed	MSD, URD	TCR	KIR B/x donor: higher aGVHD^3−4^ (more evdient in HLA mismatched transplants)
Neuchel et al. (72)	1446	Mixed	URD	TCR	KIR2DS2: higher OS and DFS (HLA-C2C2 patients); KIR2DS1: lower relapse but higher TRM (HLA-C2C2 patients); KIR2DS5: lower relapse (HLA-C2C2 patients)
Gaafar et al. (74)	87	Mixed	MSD	TCR	KIR2DS2: HLA-C1 match: higher aGVHD^2−4^ (AML); KIR2DS1: HLA-C2 match: higher cGVHD (AML); Donor presenting KIR2DL1 or 2DS2: higher cGVHD (AML)
Sahin et al. (95)	96	AML, CML	MSD	TCR	KIR B/x donor: higher cGVHD
Heatley et al. (96).	152	Mixed	MSD	TCR	KIR2DS2: higher OS (AML); Cen-B/x donor: higher OS (AML) and lower aGVHD^2−4^ (AML); Tel B/x donor: lower CMV reactivation
Babor et al. (97)	317	Pediatric ALL	MSD, URD	TCD#, TCR	Higher ct-KIR score: lower relapse
Tordai et al. (98)	314	Mixed	MSD, URD	/	The combination of KIR2DS1 donor with HLA-C2 pos patients: higher OS
Nakamura et al. (99)	288	AML	MSD, URD	TCD^*^, TCD#	CMV reactivation: lower relapse and higher NRM (more evident in KIR B/x donor or when donor presenting KIR2DS1)
Bultitude et al. (100)	119	AML	URD	TCD, TCR	Cen-B/x donor: lower OS and NRM, higher IRM
Weisdorf et al. (101)	2662	AML	URD	TCD#, TCR	KIR B/x donor: lower relapse and higher LFS (RIC)
Verneris et al. (46)	716	Pediatric AL	URD	TCD#, TCR	KIR gene content: no significant impact on OS, DFS, relapse, TRM, or aGVHD
Zhao et al. (49)	64	Mixed	HRD	TCD#	KIR2DS3: higher aGVHD and cGVHD; KIR2DS5: higher aGVHD
Symons et al. (102)	86	Mixed	HRD	TCD#	KIR B/x donor: lower NRM and higher OS, EFS (KIR AA patients)
Chen et al. (76)	84	Mixed	HRD	TCD#	KIR2DS2: higher OS (lymphoid cohort); KIR2DS1: higher GVHD (lymphoid cohort)
Michaelis et al. (50)	57	Mixed	HRD	TCD^*^	KIR B/x donor: lower relapse
Zhao et al. (77)	97	CML	HRD	TCD#	KIR2DS3: lower EFS and OS, higher TRM; KIR2DS5: higher EFS and OS, lower TRM; KIR B/x donor: higher aGVHD^3−4^
Oevermann et al. (103)	85	Pediatric ALL	HRD	TCD^*^	KIR B/x donor: lower relapse and better EFS; High donor KIR-B content: lower relapse and better EFS
Mancusi et al. (51)	161	AML, ALL	HRD	TCD^*^ TCD^*^+Treg/Tcon	Tel B/x vs. Tel AA: lower NRM and higher EFS (NK-alloreactive donors) KIR2DS1/3DS1: lower NRM and higher EFS (NK-alloreactive donors) KIR 2DS1 binding to HLA C2: increased inflammatory cytokine
Zhao et al. (53)	180	Mixed	HRD	TCD#	KIR2DS2: higher CMV reactivation
Solomon et al. (79)	208	Mixed	HRD	TCD#	KIR B/x donor with 2DS2 vs. KIR B/x donor without 2DS2: higher OS and DFS, lower relapse and NRM; KIR B/x donor with 2DS2 vs. KIR A/A donor: higher OS and DFS, lower NRM
Perez-Martinez et al. (104)	192	Pediatric mixed	HRD	TCD^*^, TCD#	KIR AA donor: higher relapse and lower DFS

*pos: positive; neg: negative; NHL, non-Hodgkin lymphoma; PFS, progression-free survival; NRM: non-relapse mortality*.

TCD^*^: ex-vivo TCD; TCD^#^: in-vivo TCD.

## KIR and Transplant Outcomes

### NK Cell Alloreactivity and GVHD

GVHD is an important complication of HSCT with high morbidity and mortality in which allogeneic donor immune cells are activated by APCs and then recognize and attack the host tissue (105). Removing donor T cells from grafts reduces the occurrence of GVHD, while it also elevates the risk of graft failure and disease relapse (106–108).

As another component of immune cells, previous murine studies suggested that adoptive transfer of interleukin-2 (IL-2)-activated SCID NK cells with donor bone marrow cells promoted engraftment in allogenic hosts with no signs of GVHD (109). Later, Asai et al. reported that hosts receiving MHC-incompatible bone marrow and spleen cells (as a source of T cells) rapidly succumbed to acute GVHD, while hosts who additionally received IL-2-activated donor NK cells on day 0 experienced a significant improvement in survival because of the lower incidence of severe GVHD. They further demonstrated that that the protective effect on GVHD was dependent on the transforming growth factor-beta (TGF-β) and could be abrogated by an anti-TGF-β antibody (35). Moreover, Ruggeri et al. showed that pre-transplant alloreactive Ly49 (Ly49 receptors recognize major histocompatibility complex (MHC) class I molecules in mice, which is analogous to KIR in humans) ligand-mismatched donor NK cell transfusion successfully eliminated host tumor cells and protected against GVHD by depleting host APCs. In contrast, hosts receiving bone marrow grafts without NK cell infusion died of GVHD, and non-alloreactive Ly49 ligand matched NK cell infusion did not provide protection against GVHD (36). Consistently, subsequent studies also found that donor alloreactive NK cells suppressed GVHD by inhibiting T cell proliferation and activation (37, 110). However, the protective role of NK cells in GVHD pathogenesis has also been challenged. Pre-clinical evidence from a xenogeneic model showed that an *in vitro* IL-2-activated human NK cell infusion promoted GVHD in SCID mice via the production of cytokines such as IFN-γ and tumor necrosis factor-α (TNF-α) (111, 112). Accordingly, GVHD was inhibited after the administration of anti-IFN-γ and depletion of Poly I:C-activated NK cells in murine studies (113, 114).

In patients with hematological malignancies, a purified (115, 116) or cytokine-induced (117–121) donor NK cell transfusion was also well tolerated and seldom induced severe GVHD (grade III-IV acute GVHD or moderate-to-severe chronic GVHD). More recently, a pilot study suggested that, after haplo-HSCT, patients with refractory AML who received a donor NK cell infusion experienced a significantly lower grade II-IV GVHD than did those without NK cell infusion (122). In contrast, Shah et al. observed that patients who received a donor IL-15/4-1BBL-activated NK cell infusion after T cell-depleted (TCD) stem cell transplantation experienced a high risk of GVHD (123).

In addition to the technique of adoptive transfer, many studies have analyzed the effects of innate donor-recipient NK cell alloreactivity on GVHD in a clinical setting. The majority of studies did not report a significant association between these parameters (41–44, 46, 47, 50, 51, 54–56, 59, 65, 66, 79, 81, 83, 87–89, 91–93, 97, 98, 102, 104), while some reported a protective effect (70, 74, 76). Moreover, several studies found that KIR ligand mismatch or receptor-ligand mismatch increased the risk of GVHD (45, 57, 60, 64, 68, 80). Accordingly, two studies performed in China that applied the ‘Peking protocol’ for HSCT using the granulocyte-colony stimulating factor (G-CSF)-mobilized graft containing a high dose of T cells observed promotive effects of NK cell alloreactivity on GVHD (48, 49).

It is not entirely clear why the reconstituted alloreactive NK cells were unable to prevent GVHD as the adoptively transferred NK cells. Studies have indicated that this discrepancy was probably attributable to the impaired function of early reconstituted NK cells. Shilling et al. first observed that a period of several months or even years was required for the recipient to reconstitute an NK cell repertoire resembling that of the donor (124). Vago et al. also suggested that the NK cells that were reconstituted early after transplantation were immature and exhibited compromised cytotoxicity (125). In addition, NK cell reconstitution is affected by graft composition. Patients receiving more T cells in grafts experience a faster T cell reconstitution (126, 127), while the absolute number of reconstituted NK cells and KIR expression are impaired by the co-grafted T cells (127–130). Other than NK cells, nearly 5% of CD8^+^ T cells, 0.2% of CD4^+^ T cells, and 10% of γδ T cells in the peripheral blood also express KIRs (131–133). Therefore, it is possible that the potential beneficial effects of alloreactive NK cells are overwhelmed by the strong alloreactive T cell response. In addition, it was observed that NK cells generated more IFN-γ in the presence of T cells in grafts, leading to a higher occurrence of acute GVHD (aGVHD) (130). Moreover, post-transplant immune suppression also exerted negative effects on NK cell reconstitution (134, 135).

Regarding specific genotypes, some studies have reported that KIR haplotype B donors afforded a significantly reduced risk of GVHD (60, 63, 86, 96). Consistent with these findings, Sivori et al. suggested that donor NK cells expressing KIR2DS1 were efficient in killing allogenic dendric cells in the setting of haplo-HSCT, thus leading to a better GVHD control (136). However, several studies also found that donors with KIR-B/x led to higher GVHD occurrence in recipients compared with donors with A/A, probably because of the more potent production of IFN-γ by alloreactive NK cells (40, 45, 76, 77, 94, 95).

Other factors, such as HLA mismatch, disease type, patient age, GVHD prophylaxis, and graft source, were also reported to interfere with GVHD occurrence in these studies (44, 45, 63, 66, 87, 92, 93, 104). Collectively, the manner in which the reconstituted NK cells affect the risk of GVHD remains largely unknown, and the relationships between NK and T cells during the initiation and process of GVHD warrant further investigation.

### NK Cell Alloreactivity and Infection

Infections are especially challenging for patients after HSCT because of the immunological derangement caused by multiple factors, including an intensive conditioning regimen, immunosuppressive agents, and other complications, such as GVHD (137, 138).

Several studies have reported that patients receiving KIR ligand-mismatched transplants are more vulnerable to infections. Schaffer et al. first reported that KIR ligand mismatch was associated with an increased infection-related mortality (43). Similarly, results from Zhao et al. showed that recipients from the KIR ligand-mismatched group experienced a significantly higher cytomegalovirus (CMV) reactivation rate. Moreover, the percentage of interferon-gamma (IFN-γ)-expressing NK cells in the peripheral blood was significantly higher in the KIR ligand matched group 30 and 100 days post-HSCT compared with the KIR ligand-mismatched group (53). The higher level of IFN-γ secretion from the NK cells might trigger Th1 immune responses, antigen presentation cell activation, and macrophage killing (7, 8), leading to lower infection rate. While, KIR ligand mismatch may increase the risk of infection by eliminating recipient APCs by donor alloreactive NK cells (36).

Many studies have found that KIR-B genes protect patients with HSCT against infections and most of them were predominantly T cell replete (TCR) transplants (81, 84, 87, 96, 139, 140). Cook et al. first observed that KIR haplotype B donors exhibited a significant reduction in the rate of CMV reactivation in sibling allo-HSCT (139). Wu et al. and Zaia et al. reported that donors expressing higher numbers of activating KIRs were associated with a lower CMV reactivation rate (62, 84). Specifically, activating KIR2DS2 and KIR2DS4 may play a major protective role (84, 140). Importantly, transplantations from donors with KIR2DS1 correlated with better infectious control (51, 96). Mancusi et al. further demonstrated that the binding of KIR2DS1 to HLA-C2 triggered pro-inflammatory cytokine production by alloreactive NK cells (51). Moreover, without a cognate ligand (HLA-C1) in recipients, donor KIR2DS2 was associated with a higher CMV reactivation rate after HLA-identical sibling HSCTs (81). Apart from CMV reactivation, the incidence of bacterial infections was also reduced when patients had KIR-B/x donors (87). In contrast with previous results, KIR2DS2 gene and Cen-B/x donors related to a higher incidence of CMV reactivation and infection-related mortality in TCD transplants (53, 100). The reasons for these differing results may be due to the different graft composition. As previously described, NK cells generate more IFN-γ in TCR transplants, which may benefit the infection control (130). Of notice, the activating KIR targets outside of HLA are largely unknown, and these clinical observations still need to be confirmed by definitive functional analysis in the future.

### NK Cell Alloreactivity and Relapse/Survival

Primary disease relapse remains the main obstacle that hampers the long-term survival of patients with hematological malignancies. Previous experience showed that adoptive transfer of autologous NK cell for patients with tumors was safe but inefficient (141–145), probably because autologous NK cells could not overcome the inhibition mediated by tumor cells expressing self-HLA. In contrast, allogenic (117), especially haploidentical, donor NK cell infusion demonstrated wide prospects in the salvage treatment (115, 120, 121) and prophylactic treatment (118, 119) of patients with hematological malignancies. In allo-HSCT, whether the reconstituted alloreactive NK cells prevent the disease relapse remains controversial.

In HLA-mismatched transplants, the Perugia group first observed that, in the context of T cell depletion, high stem cell dose, and absence of post-transplant immune suppression, KIR ligand mismatch reduced the risk of relapse and markedly improved survival in patients with AML, but not in those with acute lymphoblast leukemia (ALL) (36). This protective effect on relapse or survival was supported by many clinical studies (42, 44, 47, 51, 54), especially in myeloid disease (44, 47, 51) and transplants with TCD grafts (42, 47, 51, 54). However, conflicting results stemmed from many studies that failed to replicate these results (39, 46, 58, 102), and some even reached the opposite conclusions (41, 43, 45, 48, 50, 55).

Studies using the receptor-ligand model including HLA-matched donor-recipient pairs also reported conflicting results. Leung et al. first reported that the receptor-ligand model was more accurate than the KIR ligand model when predicting the risk of relapse, especially for lymphoid malignancies. Moreover, the potency of the relapse protection positively correlated with the number of receptor-ligand mismatch pairs (39). Subsequently, the protective effect of receptor-ligand mismatch has been confirmed by many investigations (58, 59, 66, 69, 71, 73, 76, 79, 80). Moreover, a survival advantage was also observed in patients with receptor-ligand mismatch compared with receptor-ligand matched pairs (59, 66, 69–71, 73, 76, 79). However, several other studies described opposite results (63, 64, 75, 77, 78). Of notice, two studies from Japan observed that the lack of the HLA-C2 ligand for donor inhibitory KIR afforded relapse protection in patients with AML and chronic myeloid leukemia, but increased the relapse rate in patients with ALL (73, 75). To date, no plausible explanation has been put forward for this disparity in relapse.

In contrast to the controversial results described above, transplantations from KIR haplotype B donors achieved greater agreement. Cooley et al. observed that patients with AML with KIR-B/x donors experienced a 30% improvement in RFS compared with those with A/A donors (40). Subsequently, many further investigations confirmed this beneficial effect of the KIR-B haplotype on relapse and survival in patients with hematological malignancies (50, 51, 57, 67, 72, 76, 79, 81, 85, 88–92, 96, 98, 101–104). Five of these studies reported that the protection effects mainly existed in the KIR Cen-B locus (67, 88, 91, 92, 96). Babor et al. further suggested that the presence of Cen-B with absence of Tel-B improved leukemia control in pediatric patients with ALL (97). At the genetic level, the KIR2DS2 gene, which is located on the Cen-B motif (72, 76, 79, 90, 92, 96), and the KIR2DS1 gene, located on the Tel-B motif (51, 72, 82, 98), were found to be related to a decreased relapse rate or an improved survival. However, several studies found that Cen-B donors indicated a lower OS (56, 70, 100). Meanwhile, Verneris et al. did not find any association between transplant outcomes and NK cell alloreactivity or KIR gene content in pediatric patients with acute leukemia (46).

Recently, Krieger et al. developed a scoring system, in which interactions of multiple KIR genes and HLA ligands were quantitatively analyzed. This comprehensive method raised an improved strategy to select a donor and exhibited great potential in the future (146).

Collectively, it is still controversial to determine an optimal donor who exhibits the best NK cell function using the three established KIR models. A better knowledge of NK cell reconstitution after HSCT may promote a better understanding of how NK cells affect the transplant outcomes in these patients. More in-depth studies focusing on “functional changes in NK cells” rather than “match or mismatch” may help us get closer to an optimal donor.

## NK Cell Reconstitution After Transplantation

### Maturation and Differentiation of NK Cells

NK cells are derived from the CD34^+^ hematopoietic stem and precursor cells in the bone marrow, which then migrate to the periphery (147). Recent evidence suggested that not only the bone marrow, but also secondary lymphoid tissues contribute to the development of NK cells (148). According to the surface expression of CD56, NK cells could be divided in two main subtypes: CD56^bright^ and CD56^dim^ NK cells. CD56^bright^ NK cells exist mainly in lymph nodes and tonsils, while CD56^dim^ NK cells, the more mature subset transformed from CD56^bright^ NK cells, are dominant in the peripheral blood (7, 147, 149, 150). CD56^bright^ and CD56^dim^ NK cells are equipped with distinct functions. The former population responds rapidly to interleukin-mediated stimulation with proliferation and cytokine secretion, while the latter population displays higher cytolytic capacity and lower proliferation (7, 8, 149). During the process of maturation, CD94/NKG2A is the first receptor that is expressed on immature NK cells. Together with the downregulation of CD56 expression, NK cells upregulate CD16 expression, lose NKG2A, and acquire KIR receptors. Finally, a subset of CD56^dim^ cells continue to differentiate and express CD57, together with an increased KIR expression and a completely abolished proliferative ability (150, 151).

In HSCTs with post-transplant cyclophosphamide (PT-Cy) as GVHD prophylaxis, NK cells experience two waves of expansion. After graft infusion, peripheral NK cells and T cells (mainly mature cells from the donor) were detectable at very low levels. PT-Cy administration results in a further decrease in T cells and NK cells, and NK cells are barely detectable in the peripheral blood. Subsequently, the reconstituted NK cells gradually recover and express high levels of CD56 and NKG2A. Around 60 days after transplantation, the KIR expression returns to normal. The expression of CD56 and NKG2A gradually decreases and becomes stable at 9–12 months post-transplantation. Other receptors expressed on NK cells, such as DNAM-1and 2B4, also require several months to return to normal (152). In summary, post-transplantation NK cell reconstitution is a long-term process (124, 125, 152).

### KIR Education: From Anergic to Responsive

As described earlier, the random combination of KIR receptor and HLA ligand can exist in healthy individuals. However, the autoimmune attack is inhibited because each NK cell expresses at least one self-inhibitory receptor. To avoid autoreactivity, NK cells must undergo an education process: NK cells expressing inhibitory KIR for self-HLA ligand (self-KIR) are educated, which means that these cells can be inhibited by self-inhibitory signals and become alloreactive against self-HLA-deficient targets. In contrast, NK cells expressing an inhibitory KIR that lacks a self-HLA ligand (non-self KIR) are uneducated, which means that they are tolerant to the self but also to infected or malignant cells (19, 21).

**Figure 3:**
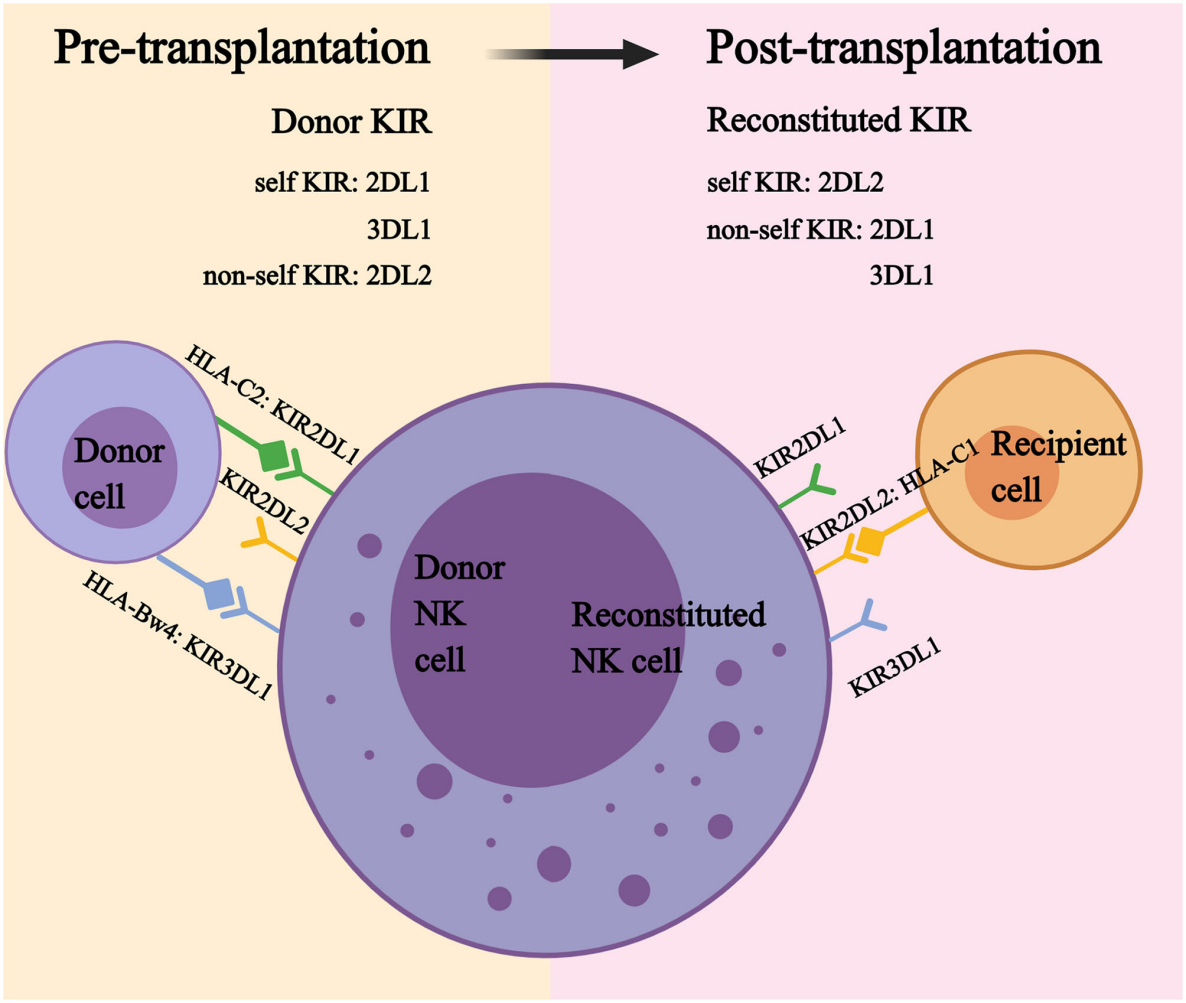
Self KIR and non-self KIR.

In the last decades, studies on KIR education have much extended our knowledge of NK cell function. After transplantation, most reconstituted NK cells express a donor-like KIR repertoire that is significantly different from that of recipient NK cells prior to transplantation (124, 151). Therefore, reconstituted NK cells expressing donor KIR may exert alloreactivity in recipients, or become anergic, as recipients may not present the cognate HLA (Figure 3). Foley et al. and Björklund et al. observed that reconstituted NK cells with non-self KIR remained tolerant, while those with self KIR acquired better functions after transplantation (65, 153). However, Yu et al. reached the opposite conclusion that alloreactive NK cells broke the self-tolerance and displayed functional capacities in the first 3 months, then gradually acquired self-tolerance by day 100 post-transplantation (154). Rathmann et al. also suggested that alloreactive NK cells were increased in the peripheral blood and exhibited a GVL effect in the early period after transplantation (155). One possible explanation for this observation is that the infusion of a megadose of donor CD34^+^ cells may create a transient donor dominant HLA environment in recipient bone marrow, and the early reconstituted NK cells expressing non-self KIR for the recipient may become educated by donor HLA and acquire functions (156). After migration to a recipient-dominant environment, reconstituted NK cells may gradually lose their responsiveness.

In murine studies, it was observed that mature NK cells from major histocompatibility complex (MHC) class I-sufficient mice become hyporesponsive after transfusion into MHC class I-deficient mice. Conversely, anergic NK cells from MHC class I-deficient mice acquired functions after exposure to the MHC class I-sufficient environment (157, 158). Using a murine transgenic model of HLA-B^*^27:05 exhibiting the Bw4 ligand for KIR3DL1, Boudreau et al. observed similar results in stem cell transplantation. CD34^+^ cells from KIR3DL1^+^ donors were transfused to B27 Tg^+^ and Tg^−^ mice, respectively. A functional analysis suggested that the most cytotoxic responsive cells were KIR3DL1^+^ NK cells from Bw4^+^ donors and developed in B27 Tg^+^ mice (Bw4^+^ donors and Tg^+^ mice), while the least-responsive cells were KIR3DL1^+^ NK cells from Bw4^−^ donors and developed in Tg^−^ mice (Bw4^−^ donors and Tg^−^ mice). Recipients with the other two combinations (Bw4^+^ donors and Tg^−^ mice and Bw4^−^ donors and Tg^+^ mice) displayed a medium level of responsiveness. The stepwise escalation of NK cell responsiveness suggested that both the donor and recipient MHC environments are critical for the maintenance and adjustment of NK cell education (159).

Recently, the Nowak team proposed that inhibitory KIR (iKIR)-HLA pairs could predict the post-HSCT NK cell education status, i.e., donors presenting cognate HLA for donor iKIR and recipients lacking it predict a downward education level; in contrast, recipients presenting cognate HLA for donor iKIR and donors lacking it predict an upward education level. Those authors found that the decrease in iKIR–HLA pairs post-transplantation is associated with a higher relapse and poorer survival (160–162), indicating that reconstituted NK cells acquire better functions after interaction with more cognate HLA class I ligands in recipients. Zhao et al. also observed that, when the donors and recipients expressed three major HLA ligands (HLA-C1, C2, Bw4), patients with AML and myelodysplastic syndrome (MDS) experienced the lowest relapse rate, and NK cells expressing three inhibitory receptors exhibited the greatest cytotoxicity and cytokine responsiveness against K562 targets (163).

Based on the findings described above, it is likely that three factors (donor KIR, donor HLA, and recipient HLA) all contribute to the variation in NK cell function. Therefore, the KIR ligand and receptor-ligand models, which only take two factors into account, may not accurately predict donors that exhibit the greatest NK cell function post-transplantation.

### Factors That Affect NK Cell Reconstitution

Although CMV reactivation suggests an immune-compromised state, patients who experienced CMV reactivation had a lower relapse rate or better survival (70, 98, 99, 164). This protective effect might be attributed to the rapid maturation of NK cells. During CMV reactivation, NK cells that express NKG2C rapidly expand and continue to increase for 1 year (165). The number of CD56^dim^ NK cells in the peripheral blood, their KIR expression, and IFN-γ production in response to K562 cells were also elevated in patients who developed CMV reactivation (165–173). Furthermore, nearly 60% of NKG2C^+^ NK cells achieved complete differentiation and expressed CD57 after CMV reactivation. These cells were termed memory-like NK cells and could be detected long after the primary CMV infection, offering a long-lasting protection (147, 166). In contrast, for non-CMV-infected patients, a higher proportion of NKG2A^+^ NKG2C^−^ KIR^−^ NK cells in the peripheral blood indicates a slow NK cell maturation. Interestingly, CMV antigen exposure to recipients also leads to an increased frequency of NKG2C^+^ NK cells, accompanied by increased KIR expression and decreased NKG2A expression (174).

As mentioned above, T cells in the graft impair the recovery of NK cells and KIR reconstitution (127–130). A possible explanation for this observation is that T cells compete with NK cells for IL-15, a cytokine that regulates immune cell survival and development (175, 176). Unlike *ex-vivo* TCD grafts, pre-transplant anti-thymocyte globulin (ATG) administration results in partial T cell depletion. Two recent studies found that ATG administration promoted NK cell recovery and delayed the reconstitution of CD4^+^ and CD8^+^ T cells, while sparing the effector memory T and regulatory T cells (Tregs) (177, 178). Compared with ATG, PT-Cy is more efficient in eliminating NK cells, with a higher residual ratio of CD4^+^ T cells and Tregs (179). Of note, several studies showed that T cells in the graft may contribute to a better NK cell function (153, 180). Several studies reported that CD56^bright^ NK cells in lymph nodes could be stimulated by IL-2-producing T cells, resulting in NK cell maturation with higher IFN-γ secretion and cytotoxic functions (181, 182).

The relationship between GVHD and NK cell reconstitution remains controversial. Previous studies demonstrated that GVHD correlated with an impaired NK cell reconstitution and KIR expression (183–185). Ullrich et al. found that CD56^bright^ NK cells were dramatically decreased in patients with GVHD, while CD56^dim^ NK cells, the more mature subtype, did not show significant changes (185). In addition, Hu et al. found that the NKG2A subset of CD56^dim^ NK cells was significantly decreased in patients with GVHD. Remarkably, a functional analysis showed that NKG2A^+^ NK cells from GVHD and non-GVHD patients exhibited a comparable GVL effect. Furthermore, the co-culture of donor T cells with NKG2A^+^ cells from non-GVHD patients suggested that NKG2A^+^ NK cells inhibit T cell proliferation and activation, indicating that the decreased number of NKG2A^+^ NK cells might be a cause, rather than a consequence, of GVHD (186). In addition, the administration of immunosuppressive agents could also affect immune recovery. Both Ullrich et al. and Giebel et al. suggested that steroid treatment, rather than GVHD, was related to the delayed NK cell reconstitution (184, 187).

## Future Directions

Numerous studies have found that alloreactive NK cells affect treatment outcomes. Although great progress has been made through both pre-clinical and clinical investigations based on the three KIR models, the controversy remains, especially regarding the benefits of KIR alloreactivity on relapse control. Recent findings showed that donor KIR, donor HLA, and recipient HLA environment all contribute to the variation of NK cell function. The newly proposed iKIR-HLA pair model needs to be further examined in the future.

NK cells, the lymphocytes that are reconstituted first after transplantation, could be negatively affected by the T cells in the graft. However, NK cell function could also be promoted through T-cell-mediated activation. The exact interactions between NK and T cells, as well as the strategy to trigger a potential synergistic NK and T cell effect remains to be investigated.

It is noteworthy that the protective role of NK cell alloreactivity in relapse protection mostly exists in myeloid disease; in fact, some studies even found that NK cell alloreactivity increased the risk of relapse for patients with lymphoid disease. The discrepancy between expressing ligands among different diseases and their binding affinity to KIR should raise more attention. In this way, we might identify which patients would benefit from the KIR-based donor selection.

## Conclusion

In the early period after transplantation, reconstituted alloreactive NK cell may not directly influence GVHD occurrence, as it is immature and it could be affected by T cells and immunosuppressive agents. The compatibility between donor KIR and the recipient HLA ligand may protect patients from infection. In the late period after transplantation, the iKIR-HLA pair model may reflect the variation in NK cell function, and quantitative analysis of KIR-HLA interactions may provide more convincing results regarding relapse and survival.

## Author Contributions

YZ and HH designed. FG and YY wrote this paper. All authors revised and approved the final manuscript.

## Conflict of Interest

The authors declare that the research was conducted in the absence of any commercial or financial relationships that could be construed as a potential conflict of interest.
